# The Use of Laser Energy for Etching Enamel Surfaces in Dentistry—A Scoping Review

**DOI:** 10.3390/ma15061988

**Published:** 2022-03-08

**Authors:** Anca Labunet, Andrada Tonea, Andreea Kui, Sorina Sava

**Affiliations:** 1 Dental Materials and Ergonomics Discipline, Iuliu Hațieganu University of Medicine and Pharmacy, 400089 Cluj Napoca, Romania; labunet@yahoo.com (A.L.); an.dr_ada@yahoo.com (A.T.); savasorina@yahoo.com (S.S.); 2 Prosthetic Dentistry Discipline, Iuliu Hațieganu University of Medicine and Pharmacy, 400006 Cluj Napoca, Romania

**Keywords:** laser treatment, etch, enamel surface

## Abstract

Background: In dental practice, different situations require etching the enamel layer. Acid etching, the present golden standard, may be replaced by other methods, such as laser etching. The main focus of our scoping review is to assess the existent literature regarding the effectiveness of different types of lasers, to identify the main aspects studied so far, and to understand where new search strategies are needed. Methods: The search was conducted in several databases focusing on the laser etching of human definitive enamel. We included English language articles published between January 2000 and December 2021. Results: The 34 articles reviewed showed that hard lasers, Er:YAG, Er,Cr:YAG, may represent an alternative etching method on enamel surfaces. They create a fractured, irregular surface and open dentin tubules, highly suitable for adhesion but with a lower risk of cavity formation. Nd:YAG, CO_2_, and Diode lasers do not help in creating sufficient shear bond strength. There is, however, evidence suggesting that microcracks in the enamel layer may appear after thermomechanical ablation using laser energy. Conclusions: While the use of acid etching is still successfully used for enamel conditioning, some researchers have emphasized the role played by saliva in the enamel-remineralization process a few days after the procedure. In this context, laser energy can be used, especially for bonding ceramic brackets in the case of orthodontic treatments. However, as thermomechanical ablation can generate microcracks, further research is required in order to establish clear findings concerning the use of laser energy on enamel etching.

## 1. Introduction

Miaman [1] pioneered the application of lasers in dentistry in 1960, and, up until now, their applications have continued to expand. Based on the active medium, several types of lasers are available: (1) gas lasers, such as carbon dioxide (CO_2_); (2) solid-state lasers, such as neodymium yttrium aluminum garnet (Nd:YAG), the erbium-doped yttrium aluminum garnet laser (Er:YAG); (3) liquid (dye) lasers, such as Rhodamine G6 (containing liquid colorant as the medium); (4) semiconductor lasers, such as GaAs or GaAIAs lasers, having a semiconductor as the medium, also known as Diode lasers and (5) ‘free-electron’ lasers, which use an electron accelerator, but are not available for dental applications [1]. Diode lasers, also known as soft lasers, are considered in low-level laser therapy or ‘bio-stimulation’ [1,2]. Lasers can be used to perform a variety of dental treatments, including frenectomies, crown shaping, composite polymerization, and control of hemorrhaging, caries detection and removal, pain and hypersensitivity treatments, gingivectomy, gingivoplasty, and soft-tissue lesions’ treatment [3].

The CO_2_ laser wavelength has an important high affinity for water, thus rapidly removing soft tissue and providing homeostasis without penetrating tissues. However, its disadvantages refer to its large size, high price, and its capacity to interfere with and destroy hard tissues [4]. Erbium lasers have two distinct wavelengths, Er,Cr:YSGG (yttrium scandium gallium garnet) and Er:YAG (yttrium aluminum garnet), with the most important absorption of water in any dental laser wavelength and a high affinity for hydroxyapatite. They can be used for treating dental hard tissues [5]. The Nd:YAG wavelength is used in surgery for removing and coagulating dental soft tissues and in periodontal treatments [6].

The Diode laser has several applications in dental practice, being used frequently for crown reshaping through gingivoplasty, frenectomies, exposure of superficially impacted teeth, removal of inflamed and hypertonic tissues, and photostimulation of the aphthous and herpetic lesions [7].

There are four different possible interactions of lasers with a target tissue: reflection, transmission, scattering, and absorption. Through absorption, the laser elevates the temperature and creates photochemical effects varying depending on the water content of the tissues. Depending on the temperature reached, ablation–vaporization of the water in the tissues, denaturing of the proteins, or dehydration and burning of the tissue—carbonization—can occur. Absorption requires a molecule that absorbs light, known as *chromophores*, with an affinity for specific wavelengths of light. In the soft tissue present in the oral cavity, chromophores are melanin, hemoglobin, and water, and in hard tissues of dental origin, they are water and hydroxyapatite. There are different absorption coefficients depending on the wavelengths the lasers have [8,9].

In clinical practice, different situations (composite fillings, adhesive techniques in restorative dentistry, bracket bonding in orthodontics, etc.) imply etching the enamel layer. Acid etching involves a selective dissolution of the enamel, causing microporosities, resulting in bonding via mechanical retention. This classic technique of acid etching of the enamel layer was introduced by Buonocore [10], while Newman used this technique for bracket bonding in orthodontics by using composite resins on the etched dental surfaces [11]. 

Ever since the application of laser energy in dentistry, various laser types have been applied when etching the enamel and dentin layer. It is known that laser irradiation on the enamel layer produces melting and recrystallization, which leads to a surface roughness comparable to the one obtained after acid etching at a microscopic level [11,12].

**Figure 1:**
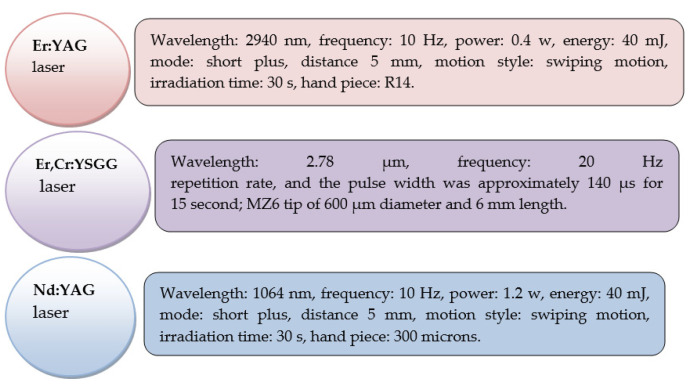
Parameters used for enamel etching, according to the laser type [11,15].

The wavelength, power, mode of operation (pulsed, continuous wave), and exposure duration all affect the results of laser applications. Irradiation was tested as a viable way to produce etching effects on hard dental tissue, as there have been various research papers discussing the best laser parameters to optimize etching applications (Figure 1) [11,12,13,14].

Among the advantages of using laser energy for enamel etching, reducing the probability of enamel damage with the reduction in the debonding force needed has clinical importance [11,16,17]. However, some disadvantages, such as undesired thermal side effects and the developments of microcracks (which might represent a starting point for carious attacks), have been reported [11,18]. The purpose of this scoping review was to assess the existent literature on the effectiveness of different types of lasers (Er:YAG, Er,Cr:YAG, Nd:YAG, CO_2_) on the enamel layer, to identify the main aspects studied so far regarding the topic, as well as to understand where new search strategies are needed. Our research examines the current state of science regarding lasers for different clinical situations necessitating the etching of the enamel layer and investigates any advantages of using laser energy compared to classical methods. The research question was: ’to what extent can laser energy can be used for enamel etching in dental practice?’

## 2. Materials and Methods

This study is a scoping review, considering the fact that outcomes and methodologies of studies regarding laser use for etching of the enamel layer are heterogeneous. Our research was performed according to the recommendations provided by Arkey and O’Malley in 2005, as well as the protocol guidelines provided by the Joanna Briggs Institute [19,20,21]. The search strategy was performed in accordance with the PRISMA-ScR guidelines (Figure 1) [19].

### 2.1. Search Strategy

The search included several databases—PubMed Central, Scopus, Medline via Ovid in December 2021—focusing on laser etching. Search for additional literature was completed via Google Scholar and through additional research of references from the included publications. All databases were searched between January 2000 and December 2021. The terms ’laser’, ’etch’, ’enamel layer’, and their combinations were used together using ’AND’ to build the search strategies. All references were imported and organized in the bibliographic software Mendeley^®^.

### 2.2. Selection of Articles

The inclusion criteria were as follows: all study types systematic reviews and meta-analysis, experimental studies performed on human definitive teeth, articles written in English, and articles for which full text is available. Studies performed on bovine and human temporary teeth, studies concerning the adhesion specifically on dentin layer, or research focusing on laser preparation of cavities instead of laser etching were excluded (Table 1).

**Figure 2:**
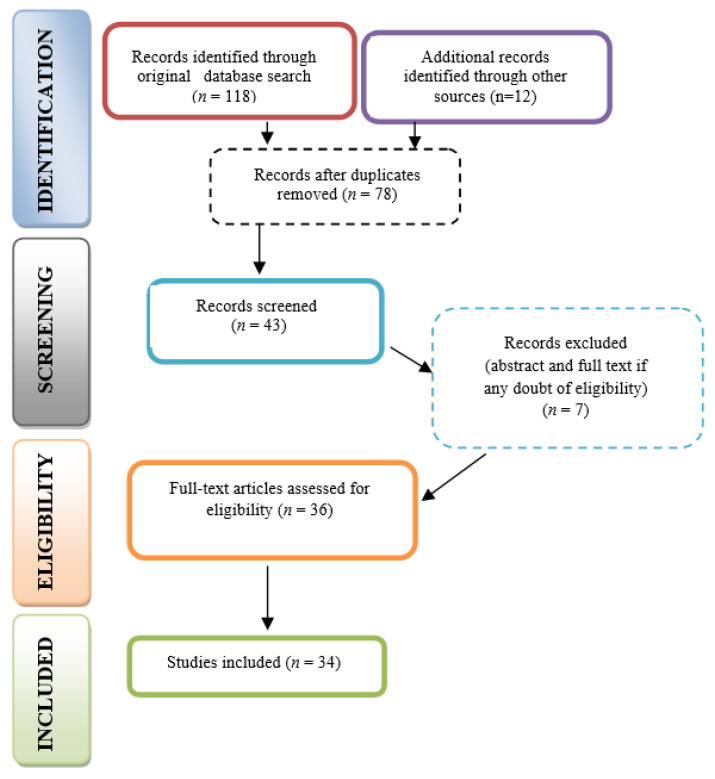
PRISMA flow diagram for research stages.

A total of 118 papers were discovered by using the search method. After the duplicates were eliminated, 78 articles were considered. The authors individually screened the abstracts in order to identify the papers that were relevant to the aims of the research, resulting in 43 studies. A total of 7 records were additionally excluded based on the outcomes, which did not match the aims of this research. After full-text reading of the resulting studies, 16 publications were eliminated because they did not meet the inclusion criteria, and a total of 34 publications were eventually included in the study (Figure 2).

### 2.3. Data Collection

From each publication, the data that were extracted included the authors, year of publication, journal, aim of study, and methodology. In addition, key findings and conclusions were also extracted. In order to organize the data, Excel spreadsheets (Microsoft Office 2019^®,^ MS, Redmond, WA, USA) were used.

## 3. Results

A selection of 34 articles was included in this scoping review. All publications investigated one of the four types of lasers—Er:YAG, Er,Cr:YAG, Nd:YAG, CO_2_—for conditioning the enamel surface. All publications are presented in three tables, according to laser types and their outcomes, in the order of publication (Table 2, Table 3 and Table 4).

## 4. Discussion

The classic method used for enamel etching is acid etching, a method that uses 37% phosphoric acid for the selective dissolution of the enamel layer, causing microporosities and resulting in a bonding mechanism via mechanical retention (the penetration of the resin tags into the microporous substrate) [11]. While this method is used successfully in different dental domains, there are some disadvantages, such as the possibility of decalcification, which leaves the enamel layer susceptible to caries attacks, as well as the discoloration caused by resin tags [11]. Although there are few studies published on enamel remineralization after acid etching using orthophosphoric acid, there is some evidence suggesting that a few days after conditioning, thanks to the role played by saliva, conditioned enamel cannot be distinguished from untreated enamel [54]. This is why novel technologies, such as laser irradiation and laser-etching techniques, have been developed as promising alternatives to acid etching. However, the use of lasers to condition the enamel surface could be even more aggressive, as it results in the ablation of similar tissue. The shear bond strength (SBS) has been investigated, especially in the context of orthodontic brackets bonding, and while some studies suggest that using self-etching primers reduces the bond strength and therefore the risk of the enamel layer fracture [55] when debonding, laser irradiation (especially Er,Cr:YSGG laser) shows promising results, especially for producing less-important adhesion forces to the enamel [56]. 

When using laser energy on the enamel surface, a melting and recrystallization process is initiated, creating a porous surface similar to the type III pattern produced by orthophosphoric acid via acid etching, thus providing an alternative to traditional etching [57]. On dental tissue, laser etching seems to create a fractured, uneven surface and open dentin tubules, highly suitable for adhesion [58].

Laser irradiation of dental hard tissues modifies the proportion of minerals in the tissues, reduces water and organic component content, and helps form stable, less-acid-soluble compounds [59]. Through this mechanism, the surface becomes less susceptible to cavity formation. Groth and collaborators found that, when combined, laser and acid conditioning increased etching depth, and laser-only etched enamel showed a small reduction in mineral concentration and higher porosity, revealing a greater penetration of acid [22].

### 4.1. Er:YAG Lasers

Most studies identified by the reviewers focused on Er:YAG lasers, a type of laser with application in cavity preparations in dental practice. Studies compared laser conditioning with conventional 37% phosphoric acid, revealing that the two methods can provide similar shear bond strengths [28,31,32,35,36]. There are certain studies revealing even a higher bond strength for laser etching [30,33]. The laser helps in improving shear bond strength values when bonding orthodontic brackets to the enamel surfaces by using a self-etching adhesive system [33]. Nd:YAG and Diode lasers also help improve the adhesion of self-etching systems in cavities [15]. When comparing Er:Yag to Nd:Yag for laser etching, the latter showed significantly lower results [25,37]. When combining acid and laser etching, the fissure sealant retention was improved [34]. 

However, three pieces of research revealed that laser conditioning of the enamel layer was less effective than acid etching [23,26,27]. These articles seem to be biased by the protocol used, lower power of lasers, or misuse of the systems. Compared to the nine studies showing improvement of adhesion when using laser, the outcome of the latter studies may be caused by technical inaccuracies.

Other studies compared different adhesive systems with significantly different results. Prime & Bond NT completely sealed dental hard tissue margins, while Etch & Prime 3.0 has shown the poorest overall results, which are statistically significant [24]. Additionally, additional laser conditioning after phosphoric acid etching might be beneficial to generation V, total etching in 2 steps [29]. 

These findings show that Er:YAG laser etching may function as a less-aggressive, high-efficacy method to create micro retention in total or self-etching adhesive systems, minimizing thermal damage. Nevertheless, thermomechanical ablation caused by these lasers can generate microcracks in the enamel layer [11].

### 4.2. Er,Cr:YSGG Laser

An Er,Cr:YSGG laser also helps increase surface roughness and eliminate the smear layer without cracking, as shown by scanning electron microscopy [38]. Similarly, 1.5- and 2-W laser irradiation may be an alternative to conventional acid etching [39,41]. Similar results between conventional acid etching and laser etching with this laser type were proven by several studies [42,44,45,46,47]. There is, however, the risk of enamel damage due to thermomechanical ablation, which can lead to microcracks [11].

Lower adhesion when using a laser was found in some studies, but the laser output was generally lower, as the main cause for such findings [40,43,48].

For this type of laser, the efficacy of etching seems slightly lower, but the advantages shown in the elimination of acid etching side effects show that it may be a viable alternative. 

### 4.3. Other Lasers

Goswami and his team studied the Nd:YAG-laser-etched enamel surface, finding a surface that is similar in aspect to other laser types but with a lower shear bond strength than acid etching [50]. One study found similar effects of bond strength when comparing Nd:YAG to acid etching [52]. Contrary to this, Fuhrmann and collaborators found CO_2_ and Nd:YAG lasers produced sufficient modification of enamel for bracket bonding [49]. However, the CO_2_ laser was proven to produce lower adhesion in most studies [51,53].

As shown by the research included in this review, Nd:YAG and CO_2_ lasers may produce similar results and are not a viable alternative to conventional etching. Their effects on hard tissue are limited, and acid etching is preferred.

## 5. Conclusions

While classic acid enamel conditioning provides suitable results in order to assure proper adhesion in dental procedures, laser use has also been considered in this matter. While there are few pieces of research on enamel remineralization after acid etching, there is evidence suggesting that, due to saliva, the conditions of enamel cannot be distinguished from untreated enamel a few days after the procedure. On the enamel surface, hard lasers such as Er:YAG and Er,Cr:YSGG seem to create a surface suitable for composite materials’ adhesion. Studies testing this method on orthodontic brackets, dental sealing, and composite fillings show mostly similar or higher adhesion than the golden standard, orthophosphoric acid. Laser irradiation of dental hard tissues helps form stable, less-acid-soluble adhesion, also lowering the risk of cavities’ formation and eliminating the smear layer. When combined, laser and acid conditioning increase etching depth. Additionally, when laser etching is prior to a self-etching adhesive, studies have shown higher shear bond strength values. Differences between findings may be caused by the laser output and power- or user-related inconsistencies. There are, however, some concerns regarding laser etching related to thermomechanical ablation, which might generate microcracks in the enamel layer. CO_2_, Diode, and Nd:YAG lasers have not been researched enough to provide us with a definitive conclusion.

While surface modifications have been thoroughly researched through scanning electronic microscopy, new-generation materials’ interaction with laser-etched surfaces may need further research. Therefore, based on the limitations of this scoping review, the results suggest that laser energy is beneficial in addition to acid etching in order to increase shear bond strength in dental-adhesive techniques. 

Based on the information provided by the studies included in this research, a possible use of laser etching in dental practice could invovle bonding ceramic brackets in the case of orthodontic treatments. As the procedure for debonding the brackets (at the end of the treatment) can produce damage to the enamel layer, the situation may require a lesser shear bond strength. 

## Figures and Tables

**Table 1:** Inclusion and exclusion criteria.

Criterion	Inclusion	Exclusion
Time period	Publications available between January 2000 and December 2021	All publications published before January 2000
Language	English	Non-English
Type of articles	All research types including primary research (e.g., experimental studies performed on human teeth, clinical trials, pilot studies), secondary research, reviews, related to the use of laser on etching of enamel layer; Publications for which full-text is available	Studies performed on bovine teeth and temporary human teeth; Studies investigating the adhesion specifically on dentin layer; Research only focusing on laser preparation of cavities

**Table 2:** Publications investigating Er:YAG laser for enamel etching.

No.	Year of Publishing	Laser Type	Methods	Results
1	[22]/2001	Er:YAG	X-ray microtomography	Laser and acid conditioning increased etching depth; laser-only etched enamel subsurface revealed small reduction in mineral concentration and increase in porosity, allowing greater penetration of acid
2	[23]/2002	Er:YAG	Total-etching adhesive OptiBond FL +/− acid etching/self-etching adhesive Clearfil SE Bond +/− laser etching	Laser etching—less effective than acid-etching
3	[24]/2002	Er:YAG	Cavities conditioned with short pulsed Er:YAG laser (500 mJ/5 Hz) + for additional 30 s using lower dosimetries (120 mJ/4 Hz): acid etch + Bond-1/acid etching+Prime & Bond NT/self-etching Etch & Prime 3.0	Prime & Bond NT completely sealed both margins; Etch & Prime 3.0 had the poorest overall results, with a statistically significant difference
4	[25]/2003	Er:YAG, Nd:YAP	37% phosphoric acid/Er:YAG laser/Nd:YAP laser	Morphological changes on hard dental tissues—higher with acid-etch and Er:YAG laser than Nd:YAG laser. Free surface energy—sgn greater with acid-etch or Er:YAG laser
5	[26]/2004	Er:YAG	37% H_3_PO_4_, diamond bur/37% H_3_PO_4_, Er:YAG laser/Er:YAG laser and 37% H_3_PO_4_/air abrasion/air abrasion + acid etching with 37% H_3_PO_4_ + compomer fissure sealant	Conventional acid etching—significatly higher sealant retention than Er:YAG laser etching or air abrasion. Mechanical conditioning of the laser or air abrasion + acid-etching results statistically equal to the acid-etch only group
6	[27]/2005	Er:YAG	Er:YAG laser and acid etching/Er:YAG laser, laser etching and acid etching/Er:YAG laser and only laser etching/high-speed bur and acid etching	Acid-etch is favored when using resin composite in Er:YAG-lased Class V cavities; the contact seaL at enamel margins in Er:YAG-lased and laser-etched cavities depended on the resin composite composition and on the adhesive
7	[28]/2012	Er:YAG	37% phosphoric acid, Er:YAG laser at 1 W/1.5 W	Mean SBS obtained with Er:YAG laser at 1 W or 1.5 W is similar to conventional etching
8	[29]/2013	Er:YAG	Total etching—3 steps and 2 steps, self-etching—2 steps and 1 step	Additional laser etching after phosphoric acid etch–beneficial to generation 5, total etching in 2 steps. No significant change or detrimental effect to the other types in SBS
9	[30]/2013	Er:YAG	Er:YAG laser + Optibond FL,/Er:YAG laser + 35% phosphoric acid + Optibond FL/Er:YAG laser + Clearfil SE Bond/a35% phosphoric acid + OptibondFL/Clearfil SE Bond	Er:YAG laser for conditioning followed by different dentin adhesive systems had an influence on the marginal sealing of composite resin restorations
10	[31]/2014	Er:YAG	Diamond bur + acid etch/cavity conditioning by Er:YAG laser + acid etch/Er:YAG laser + laser etching/diamond bur + laser etching/Er:YAG laser with no conditioning procedure	No significant difference in evaluating microleakage degree of cavities prepared by Er:YAG laser and diamond bur
11	[32]/2015	Er:YAG	Er:YAG/air abrasion/self-etching adhesive with phosphoric acid or laser/acid etching	Conventional acid etching sgn lower microleakage, higher unfilled area proportions than the Er:YAG laser + self-etch adhesive group
12	[33]/2016	Er:YAG	Acid + Transbond XT/Er:YAG (100 mJ or 200 mJ 10 Hz) etching + Transbond XT/Transbond Plus self-etching primer/Er:YAG (100 mJ or 200 mJ, 10 Hz) etching + Transbond Plus SEP/Clearfil Protect Bond/Er:YAG (100 mJ or 200 mJ, 10 Hz) etching + Clearfil Protect Bond	Lowest SBS values were in 1 step self etch; the highest were in laser+ self-etch. When two-step self-etch adhesive is used for bonding brackets, laser etching at 1 W (100 mJ, 10 Hz) seems to improve SBS
13	[34]/2017	Er:YAG	Phosphoric acid etching/Er:YAG laser + acid etching, fissure sealant	Er:YAG laser + acid etching sgn improves fissure sealant retention over conventional acid etching alone
14	[35]/2018	Er:YAG	37% phosphoric acid, Er:YAG laser and phosphoric acid etching, and combination with Er:YAG laser	Er:YAG laser and the resin composite; the resin-modified glass ionomers and fissure sealant may provide a variant of hard dental tissue etching to acid etching
15	[36]/2018	Er:YAG	37% phosphoric acid/Adper Single Bond 2; Er:YAG laser/37% phosphoric acid/Adper Single Bond 2; Clearfil SE Bond; Er:YAG laser/Clearfil SE Bond; Adper Easy One; Er:YAG laser/Adper Easy One	Er:YAG laser conditioning may show some advantage prior to Adper Single Bond 2 application in enamel
16	[15]/2019	Nd:YAG, Er:YAG, Diode	Two-step self-etching adhesives/lasers before or after primer or bonding	Nd:YAG laser after applying the primer, Diode laser after bonding agent, may sgn improve the microtensile bond strength in two-step self-etch adhesive systems
17	[37]/2021	Nd:YAG, Er:YAG	No conditioning/Er:YAG laser (2940 nm, 10 Hz, 1.2 W)/Nd:YAG laser (1064 nm, 1.5 W, 10 Hz) + self-etching adhesive	Er:YAG laser (2940 nm, 10 Hz, 1.2 W) on cavity surface shows lower marginal microleakage of self-etch adhesive resin cement restorations compared to Nd:YAG (1064 nm, 1.5 W, 10 Hz) and no conditioning groups

Abbreviations: Er:YAG = erbium-doped yttrium aluminum garnet; Nd:YAG = neodymium yttrium aluminum garnet; SEM = scanning electronic microscopy; SBS = shear bond strength; Sgn = statistically significant.

**Table 3:** Publications investigating Er,Cr:YSGG laser for enamel etching.

	Year of Publishing	Laser Type	Methods	Results
1	[38]/2001	Er,Cr:YSGG	Er,Cr:YSGG, 37% phosphoric acid	Surface roughness was sgn higher with the laser system. Scanning electron microscopy showed that the irradiated surface produced a rough surface that was entirely lacking a smear layer, with no cracking of enamel or dentin
2	[39]/2008	Er, Cr:YSGG	Er, Cr:YSGG 0.5, 0.75, 1, 1.5, and 2 W, phosphoric acid	A more important layer of adhesive was left on the enamel surface with low-power laser irradiation. Sandblasting and low-power laser irradiation (0.5, 0.75, and 1 W) seem to not etch enamel in a way that is acceptable for orthodontic molar tube bonding, but 1.5- and 2-W laser irradiation was shown to be an alternative to conventional acid etching
3	[40]/2009	Er,Cr:YSGG	Er,Cr:YSGG 0.25 W, 20 Hz, 2.8 J/cm^2^ energy per pulse of 12.5 mJ, water delivery rate 11 mL/min	Laser conditioning significantly lowered the bond strength of several adhesive systems applied on enamel
4	[41]/2010	Er,Cr:YSGG	Er,Cr:YSGG laser/37% phosphoric acid + primer adhesive; self-etched primer + adhesive; all-in-one adhesive—single dose	Er,Cr:YSGG laser > 37% phosphoric acid. SBS of laser etched + primer/adhesive group sgn higher than 37% phosphoric acid + primer/adhesive
5	[42]/2013	Er,Cr:YSGG	Phosphoric acid etching/laser etching—clinical	The clinical performance of fissure sealants placed after acid or Er,Cr:YSGG laser etching was similar
6	[43]/2015	Er, Cr:YSGG	Laser etch/phosphoric acid etched laser + silorane adhesive system	Phosphoric acid best for SBS with Silorane System Adhesive. Non etched = laser etched
7	[44]/2016	Er,Cr:YSGG	37% phosphoric acid/Er,Cr:YSGG laser 2.78 µm, 1.5 W	Overall retention rate in acid etched—slightly higher compared to laser etched; difference statistically non-significant
8	[45]/2018	Er,Cr:YSGG	Etching with 37% phosphoric acid for 15 s, irradiation with Er, Cr:YSGG laser at 1 watt for 10 s and 20 s, and irradiation with Er,Cr:YSGG laser at 1.5 watts for 10 s and 20 s. Metal brackets were bonded with Transbond XT	1.5 W/20 s Er,Cr:YSGG laser produced comparable bond strength to acid etching; no sgn diff between laser and acid etch
9	[46]/2019	Er,Cr:YSGG	Er,Cr:YSGG laser, phosphoric acid + Scotchbond Universal/Transond XT	Highest SBS—Scotchbond Universal with laser etching; Transbond XT with acid or laser etching, and Scotchbond in self-etch mode—lowest bond strength
10	[47]/2020	Er,Cr:YSGG	Er,Cr:YSSG/phosphoric acid etching/acid etching + etch-and-rinse adhesive/self-etching adhesive—in vitro. contaminating enamel surfaces with artificial saliva + fissure sealant/contamination and repeated conditioning + fissure sealant	Re-application of Er,Cr:YSSG laser and self-etching adhesive did not have an effect on microleakage of fissure sealants. Without re-application, acid-etching + etch-and-rinse adhesive—only superior to acid-etching
11	[48]/2021	Er,Cr:YSGG	Er,Cr:YSGG, 37% phosphoric acid	The shear bond strength of composite resin bonded to hard dental tissues etched with phosphoric acid was more important than results obtained when conditioned with Er,Cr:YSGG laser

Abbreviations: Er,Cr:YSGG = erbium-doped yttrium scandium gallium garnet; SEM = scanning electronic microscopy; SBS = shear bond strength; Sgn = statistically significant.

**Table 4:** Publications investigating other lasers for etching the enamel layer.

	Year of Publishing	Laser Type	Methods	Results
1	[49]/2001	CO_2_ and Nd:YAG	CO_2_ laser/Nd:YAG laser/phosphoric acid etching	CO_2_ laser—demineralization gaps of various dimensions, Nd:YAG laser—honeycomb structures similar to acid-etch technique. CO_2_ and Nd:YAG lasers—sufficient modification of enamel for bracket bonding
2	[50]/2011	Nd:YAG	35% phosphoric acid/Nd:YAG laser 0.8 W, 10 Hz, for 10 s with 80 mJ/pulse power + bonding + composite	Under SEM, acid showed typical honeycomb appearance, and laser—bubble-like cavities. In enamel, acid etching technique showed higher SBS
3	[51]/2013	CO_2_	CO_2_ laser, phosphoric acid	Initial preparation with acid has a higher SBS value than CO_2_ laser, with higher secondary bonding. Less adhesive residue present on enamel after tooth preparation with laser following debonding
4	[52]/2017	Nd:YAG	Nd:YAG laser and 37% phosphoric acid	Comparison of the compositions demonstrated that calcium has higher percentage when exposed to laser-etching compared to acid-etching. Nd:YAG laser can be implemented for etching procedure as a replacement of the conventional technique
5	[53]/2019	CO_2_	CO_2_ laser/37% phosphoric acid/polyacrylic acid/self-etching/air abrasion	The teeth etched with 37% phosphoric acid exhibited significantly greater depth of resin penetration (15.1 µm) than self-etching and polyacrylic acid. Laser etching showed similar depth with acid etching. Air abrasion shows lowest depth of all groups

Abbreviations: SEM = scanning electronic microscopy; SBS = shear bond strength; Sgn = statistically significant.

## Data Availability

Not applicable.
